# Highlights of ASCO 2025

**DOI:** 10.1093/oncolo/oyaf336

**Published:** 2025-10-07

**Authors:** Brandon DaSilva, Alfred I Neugut, Tito Fojo, Susan E Bates

**Affiliations:** Department of Medicine, Columbia University, New York, NY 10032, United States; Herbert Irving Comprehensive Cancer Center, Columbia University, New York, NY 10032, United States; Department of Medicine, Columbia University, New York, NY 10032, United States; Herbert Irving Comprehensive Cancer Center, Columbia University, New York, NY 10032, United States; Vagelos College of Physicians & Surgeons, and the Department of Epidemiology, Columbia University, New York, NY 10032, United States; Department of Medicine, Columbia University, New York, NY 10032, United States; Herbert Irving Comprehensive Cancer Center, Columbia University, New York, NY 10032, United States; Mailman School of Public Health, Columbia University, New York NY, James J. Peters Bronx VA Medical Center, Bronx, NY, USA; Department of Medicine, Columbia University, New York, NY 10032, United States; Herbert Irving Comprehensive Cancer Center, Columbia University, New York, NY 10032, United States; Mailman School of Public Health, Columbia University, New York NY, James J. Peters Bronx VA Medical Center, Bronx, NY, USA

Once again, this year, we present highlights from the annual meeting of the American Society of Clinical Oncology, held in Chicago, with over 40 000 attendees and over 5000 oral and poster presentations. As is typical of this meeting, many studies were interesting for a variety of reasons, so we had to carefully choose what to include to share with the readers of *The Oncologist*. If desired, abstracts can be accessed at ASCO.org. Where possible, we have also provided the citation for the concurrent publication—though this was not always possible.

## Lung cancer

- **Late-breaking abstract 8008** (**LBA8008**) addressed the second-line treatment of patients with extensive small-cell lung cancer (SCLC) who have disease progression following treatment with platinum-based chemotherapy.^1^^,^^2^ Tarlatamab, a bispecific T-cell engager immunotherapy drug, showed excellent results in this setting in phase 2 trials and so received accelerated FDA approval in May 2024. Now Charles Rudin of Memorial Sloan-Kettering Cancer Center reports the results of the confirmatory phase 3 multi-national Ph3 DeLLphi-304 trial in which 309 patients with extensive SCLC with disease progression on first-line platinum-containing chemotherapy were randomized to receive either tarlatamab or second-line chemotherapy. With a median follow-up of 11.2 months, the median overall survival (OS) was 13.6 months for tarlatamab versus 8.3 months for chemotherapy (hazard ratio [HR]: 0.60, 95% confidence interval [CI]: 0.47-0.77) with an improved progression-free survival (PFS) as well (4.2 vs. 3.2 months; HR: 0.72, 95% CI: 0.59-0.88). There is a plan for priority review of tarlatamab based on the results of DeLLphi-304 later this year. Given the fact that the prognosis of SCLC remains poor and its treatment challenging, expect front-line tarlatamab development to include combinations with chemotherapy and checkpoint inhibitors, and the evolution of how to mitigate cytokine-release syndrome, a difficult, although not insurmountable complication. We must demand meaningful gains in survival with these combinations, given that they will be predictably more toxic.
- Adagrasib, a KRAS G12C inhibitor, was granted FDA approval in 2022 for the treatment of patients with locally advanced or metastatic non-small-cell lung cancer (NSCLC) whose disease had progressed following one prior therapy.^3^ The phase 2 KRYSTAL-7 study evaluated the use of adagrasib plus pembrolizumab as first-line therapy for patients with advanced/metastatic KRAS G12C-mutated NSCLC.^4^ For the 149 patients enrolled in this trial, Pasi A. Janne of the Dana-Farber Cancer Institute reported an overall response rate (ORR) of 44.3% with a median OS of 18.3 months. The latter result in all patients compares favorably with median OSs for advanced/metastatic NSCLC treated in the first-line with a combination of a checkpoint inhibitor and chemotherapy, generally around 18-22 months. Comparing the outcomes for the 95 patients who had PD-L1 scores <50% vs. the 54 patients whose scores were ≥50%, the ORRs were 35.8% (95% CI: 26.2-46.3) vs. 59.3% (95% CI: 45-72.4), respectively, with median OS values of 15.5 months vs. not reached. These results in patients with high PD-L1-expressing advanced NSCLC emulate immune checkpoint inhibitor (ICI) monotherapy, which provides significant clinical benefit, and it will require a randomized trial to discern the value, if any, of adding adagrasib to an effective ICI monotherapy regimen. On the other hand, given that there is no significant clinical benefit of ICI monotherapy for patients with advanced/metastatic NSCLC with low or no PD-L1 expression, it is hoped that adagrasib alone or in combination will improve outcomes.

## Head and neck cancer

- Little has changed for a long time in the management of locally advanced resected squamous cell carcinoma of the head and neck (HNSCC). The standard of care (SOC) has been adjuvant cisplatin with intensity-modulated radiation therapy (IMRT) for at least the last 20 years, with a 40%-45% recurrence risk at 3 years. This may change now with the results of the phase 3 NIVOPOSTOP trial, presented in the Plenary Session by Jean Bourhis of the Lausanne University Hospital in Lausanne (**Abstract LBA2**).^5^ The study enrolled 680 patients with stage III or IV HNSCC *who had undergone complete surgical resection*. Half of the subjects were smokers, and 80% were either stage IVA or IVB. All the subjects received cisplatin 100 mg q3weeks along with IMRT. The treatment group initially received one 240 mg dose and one 360 mg dose of nivolumab, 3 weeks apart, and then had 6 additional doses of nivolumab at 480 mg q4weeks after the IMRT. The control group did not receive nivolumab. With a median follow-up of 30.3 months, the 3-year disease-free survival (DFS) was 63.1% (95% CI 57%-68.7%) in the nivolumab group vs. 52.5% (95% CI 46.2%-58.4%) in the control group. Adverse events were more common in the nivolumab group. Nonetheless, this is the first time a meaningful, statistically significant improvement has been observed over the SOC in this setting. If confirmed, HNSCC will join melanoma, NSCLC, renal cell cancer, bladder cancer, and gastroesophageal (GEJ) cancers in having an approved ICI therapy in the adjuvant setting.

## Colorectal cancer

- **Abstract LBA1** presented the follow-up findings of the ATOMIC study, led by the Alliance.^6^ While ICI blockade has become SOC for metastatic deficient mismatch repair (dMMR) colorectal cancer as well as for neoadjuvant therapy for MSI-high rectal cancer, its use in the adjuvant setting for colon cancer remains unclear. In this study, presented by Frank Sinicrope of the Mayo Clinic Comprehensive Cancer Center, the investigators reported a multicenter randomized phase 3 trial in which, between 9/2017 and 1/2023, 712 patients with stage III surgically resected dMMR colon adenocarcinoma were randomized to 12 cycles of mFOLFOX6 ± biweekly atezolizumab (840 mg IV) for 1 year. With a median follow-up of 37.2 months, there was a DFS benefit in the immunotherapy group, with a 36-month DFS of 86.4% vs. 76.6% for chemotherapy alone (HR: 0.50, 95% CI: 0.34-0.72). Data on OS are not yet mature and remain an important missing endpoint. The data show DFS curves that begin to separate at 6 months and achieve a maximum difference by about 12 months, 6 months after chemotherapy was discontinued, and then remain flat with gradual increases in the HRs. This suggests additional DFS benefit from atezolizumab is not accruing and raises the possibility that the DFS benefit occurs from a transient atezolizumab support of the chemotherapy-induced blunting of immunity. If true, this may then not translate into a meaningful OS benefit. These were long-awaited and anticipated results, but do underscore the importance of OS as an endpoint, especially in colorectal cancer, a disease in which approvals have almost always been granted for OS benefits [see trifluridine/tipiracil in RECOURSE and with bevacizmab in SUNLIGHT, encorafenib + cetuximab in BEACON CRC, fruquintinib in FRESCO2/FRESCO, ramucirumab in RAISE and adagrasib plus cetuximab anticipated in KRYSTAL-10, as well as **Abstract LBA3500** below].^7–17^
- Precision medicine has continued to make more and more contributions to our management of colorectal cancer. It has been recognized for 15 or more years that colorectal cancer with a BRAF-V600E mutation, present in about 10%-12% of metastatic colorectal cancers, is a particularly aggressive and difficult-to-treat tumor.^9^ But, as with other similar poor prognostic markers, when an inhibitory drug appears for this marker, its presence may then offer a more positive outcome. Encorafenib plus cetuximab was previously established as a SOC therapy for BRAF-mutated metastatic colorectal cancer with an OS benefit.^10^  **Abstract LBA3500** presents the results of the BREAKWATER Trial, presented by Elena Elez of the MD Anderson Cancer Center.^7^^,^^8^ In this study, 637 patients with untreated metastatic BRAF-positive colorectal cancer were randomized to encorafenib/cetuximab (EC), EC + mFOLFOX, or SOC. With a median follow-up of 16.8 months, the median PFS for EC was 6.8 vs. 12.8 months for EC + mFOLFOX6 vs. 7.1 months for SOC. OS in the 3 arms was 19.5, 30.3, and 15.1 months, respectively. EC+mFOLFOX6 was statistically significantly better vs. SOC for both PFS and OS. It does appear from this study that, for patients who have metastatic colorectal cancer bearing BRAF V600E mutations, the new SOC first-line therapy should be encorafenib-cetuximab-mFOLFOX6.
- Patients with resected localized primary tumors often ask what else they could do beyond adjuvant chemotherapy to reduce their risk of tumor recurrence. **Abstract LBA3510** described a study led by Kerry S. Courneya of the University of Alberta and presented by Christopher Booth of Queen’s University that may provide an answer to that question.^18^^,^^19^ Between 2009 and 2024, investigators randomized 849 patients with resected stage III or high-risk stage II (T4 with fewer than 12 lymph nodes removed and poorly differentiated) following adjuvant chemotherapy. At 55 centers, mostly in Canada and Australia, the intervention group worked with a physical activity (PA) consultant every 2 weeks for the first 6 months with 12 mandatory exercise sessions, and with 12 recommended supervised exercise sessions in the alternate weeks. This was combined with an exercise guidebook and health education materials. The number of sessions was reduced in subsequent months, but the program continued for a total of 3 years with a goal of increasing PA by at least 10 MET hours/week. The control arm received only educational materials. Adherence appears to have been satisfactory. With a median follow-up of 7.9 years, the 5-year DFS was 80% for the exercise group and 74% for the controls (HR: 0.72, 95% CI: 0.55-0.94). The 8-year OS was 90% vs. 83% (HR: 0.63, 95% CI: 0.43-0.94). The long timeframe required for recruitment and the size of the denominator population out of which participants were recruited raise some concerns, but our instincts tell us that it’s time to get our patients exercising more.
- **Abstract 3503** was presented by Jeanne Tie of the Peter McCallum Cancer Centre in Melbourne. She reported the findings of the Dynamic-III study of stage III colon cancer patients who were found to be positive on ctDNA testing after surgery.^20^ Patients were randomized to have ctDNA-guided escalation of treatment or SOC management that was blinded to ctDNA testing, with the caveat that physicians had already selected their SOC chemotherapy regimen prior to randomization. Those assigned to the ctDNA informed management arm were eligible for escalation of therapy from single agent fluoropyrimidine to an oxaliplatin-based doublet, from 3 months to a 6 months doublet or FOLFOXIRI (clinician choice), or from 6 months of doublet chemotherapy to FOLFOXIRI.^20^ Of the patients randomized, 259 patients had positive ctDNA, with 129 patients assigned to the ctDNA-escalation management arm and 130 patients in the SOC arm. In the management-escalation arm, 115 patients (89%) had their therapy escalated, with 65 patients receiving FOLFOXIRI. Of the 130 patients in the SOC arm, 14 patients (11%) received single agent fluoropyrimidine and 112 patients (86%) received an oxaliplatin doublet. With a median follow-up of 42.2 months, the median RFS was 29.2 months for the ctDNA managed arm vs 36.8 months for the SOC arm (HR: 1.11, 90% CI: 0.83-1.48) or no statistically significant benefit. What was revealing was that ctDNA clearance in patients from both arms, at 4-8 weeks following completion of treatment was associated with improved RFS (HR: 11.1, 90% CI: 6.7-20) with a 3-year RFS of 84% for the group with ctDNA clearance and 12% for those with detectable ctDNA.

## Gastric cancer

- **Abstract LBA5** was a presentation by Yelena Y. Janjigian of Memorial Sloan-Kettering Cancer Center. It sought to improve upon the longstanding use of perioperative FLOT (5-fluorouracil, leucovorin, oxaliplatin, docetaxel) that is the SOC for resectable gastric or GEJ cancer.^21^^,^^22^ The MATTERHORN study was a double-blind phase 3 trial that investigated the addition of perioperative immunotherapy to FLOT.^23^^,^^24^ There were 948 patients with stage II-IVA untreated gastric/GEJ cancer who received 4 cycles of FLOT (2 cycles neoadjuvant, 2 cycles adjuvant) plus durvalumab or placebo followed by 10 more cycles of durvalumab or placebo q4weeks in the adjuvant setting. The median follow-up time was 31.5 months. The median EFS was not reached for the durvalumab arm and was 32.8 months for the placebo group (HR: 0.71, 95% CI: 0.58-0.86). As regards OS, there was an encouraging trend for the durvalumab arm. Described as the new SOC for resectable gastric/GEJ adenocarcinoma by the discussant, D-FLOT now provides a starting point for multiple studies that will seek to answer the role of surgery, the duration of adjuvant durvalumab, if any, especially in patients with pathologic complete responses (CRs), and numerous other questions.

## Breast cancer

- **Abstract LBA109** was a presentation by Sarah Tolaney of Dana-Farber Cancer Institute on the ASCENT-04/KEYNOTE-D19 trial studying the TROP2 antibody-drug conjugate sacituzumab govitecan (SG) + pembrolizumab in the front-line setting for patients with previously untreated (or ≥6 months since prior curative treatment), locally advanced unresectable or metastatic triple-negative breast cancer (TNBC) with a combined positive score (CPS) ≥10.^25^ In this phase 3 randomized trial, 221 patients were randomized to SG + pembrolizumab and 222 to chemotherapy plus pembrolizumab, with either paclitaxel, nab-paclitaxel or gemcitabine plus carboplatin as the “physician’s choice” chemotherapy. Seeking to address the value, if any, of replacing conventional chemotherapy with SG in combination with pembrolizumab, with a median follow-up time of 14 months, the investigators reported a median PFS of 11.2 months with the SG plus pembrolizumab group vs. 7.8 months for the investigator’s choice chemotherapy plus pembrolizumab arm (HR 0.65, 95%CI 0.51-0.84). Interestingly, the Forest plot showed SG better than gemcitabine plus carboplatin, but not better than a taxane, emulating the results of KEYNOTE-355,^26^ which found paclitaxel plus pembrolizumab but not gemcitabine plus carboplatin better than placebo plus pembrolizumab. This suggests SG was only superior to the ineffective/less effective gemcitabine plus carboplatin combination but not paclitaxel. This uncertainty was further augmented by the toxicity data that found SG, the putative targeted chemotherapy, had higher frequencies of any grade nausea (68% vs. 38%), vomiting (29% vs. 14%), diarrhea (70% vs. 29%), and alopecia (52% vs. 32%) compared with the physician’s choice chemotherapy + pembrolizumab. Although the data was not mature at the time of presentation, OS was not, nor ever likely will it be, statistically better with SG and not because some patients subsequently received SG as a single agent. Thus, in the front-line setting for patients with locally advanced unresectable or metastatic TNBC, it would appear that paclitaxel as the chemotherapy partner for pembrolizumab helps physicians and patients avoid physical as well as financial toxicities.
- **Abstract LBA4** was a presentation by Nicholas Turner of Royal Marsden Hospital in London, UK reporting the results of the SERENA-6 study. This phase 3, randomized, double-blind, placebo-controlled trial explored the use of ctDNA to identify patients with ESR1 mutations (ESR1m) after ≥6 months of front-line treatment for hormone-receptor positive (HR+)/HER2-negative advanced breast cancer to determine the benefit of switching from an aromatase inhibitor (AI) to the next-generation oral selective estrogen receptor degrader (SERD) and complete ER antagonist camizestrant at the time of “molecular progression” prior to disease progression.^27^ Of the 3256 patients who underwent ESR1m testing with Guardant 360CDx every 2-3 months, 548 patients (16.8%) were found to have an ESR1m, with 51% occurring on the first test, including 53 patients who were excluded for concomitant disease progression. In total, 315 patients were randomized to receive either camizestrant with the CDK4/6i or continuing the AI with CDK4/6i. With a median follow-up of 12.6 months, median PFS was 16 months in the camizestrant plus CDK4/6i group compared to 9.2 months in the AI plus CDK4/6i group (HR: 0.44, 95% CI: 0.31-0.60).^28^ After 38 and 47 second events in each arm, respectively, the investigators claimed “PFS2” at 1 year was 85.4% in the camizestrant plus CDK4/6i arm compared to 74.4% in the AI plus CDK4/6i arm. Although there appeared to be a PFS benefit with camizestrant, in an excellent presentation, the discussant, Angela DeMichele, noted the bias in the analysis, given patients randomized to camizestrant were afforded the benefit of an ESR1m-specific therapy prior to progression on an AI+CDK4/6i and had thus experienced a PFS3. Thus, “while ctDNA switching to camizestrant at emergence of ESR1m had met the primary endpoint of prolonging first-line PFS, the clinical utility of serial ctDNA testing as a treatment strategy for ER + MBC is not yet determined.”^28^ Given the reported benefit in the SERENA-2 trial of camizestrant over fulvestrant in patients with advanced HR+/HER2− breast cancer previously treated with endocrine therapy, and the goal of prolonging life as long as possible by getting the most out of every option, not answered by the SERENA 6 study was whether waiting for clinical rather than “molecular” progression might not be preferred before switching from an AI to a drug such as camizestrant. While not the goal of SERENA 6, this strategy emerged as that most defensible by the data.

## Dermatologic cancers

- Immunotherapy with cemiplimab or pembrolizumab is approved for use in unresectable locally advanced, and recurrent/metastatic cutaneous squamous cell carcinoma (cSCC). ASCO 2025 highlighted two similar studies looking at the use of adjuvant immunotherapy following surgery and post-operative radiation in patients with high-risk cSCC. Here, we review them together to consider their comparability and the value, if any, of their use as adjuvant therapy. Both studies had similar but not identical designs and enrollment criteria, including differences when adjuvant therapy was initiated, which may have explained the dissimilar outcomes. Specifically, differences in disease/recurrence-free survival between the immunotherapy and placebo arms were reported with cemiplimab but not with pembrolizumab. The differences were both numerical and in HRs with disease/recurrence-free survivals for the control arms that differed at 12 months but were comparable beyond 24 months. In **Abstract 6001**, Danny Rischin of the Peter MacCallum Cancer Centre in Melbourne, Australia presented the results of the phase 3 C-POST trial comparing cemiplimab (209 patients) vs. placebo (206 patients) in patients with local and/or regional cSCC.^29^^,^^30^ Patients were randomized 1:1 to cemiplimab 350 mg or placebo q3weeks for 12 weeks, then cemiplimab 700 mg or placebo q6w up to 36 weeks, up to 48 weeks total. With median disease-free survival not reached in the cemiplimab group vs. 49.4 months in the placebo arm (HR: 0.319, 95% CI: 0.199-0.511) it is clear cemiplimab delayed disease recurrence. However, in **Abstract 6000,** presented by Jenny H. Lee of Chris O’Brien Lifehouse in Camperdown, NSW, Australia, a similar benefit in recurrence-free survival was not achieved with the addition of adjuvant pembrolizumab (225 patients) compared to placebo (225 patients), clouding the picture for the use of adjuvant immunotherapy in cSCC.^31^ Poorer outcomes in the pembrolizumab group and better outcomes with placebo brought the curves closer, leading to statistically nonsignificant differences (HR: 0.76, 95% CI: 0.53-1.10), despite reductions in locoregional recurrences (13.8% vs. 25.3%) and distant metastasis (4.4% vs. 11.6%) with pembrolizumab, gains blunted to some extent by more deaths with pembrolizumab (15.6% vs. 10.7%). Of the 35 deaths reported in the pembrolizumab group, 17 occurred within 1 year of randomization and none were noted to be treatment-related, but as highlighted by the authors, these early deaths likely minimized the disease-specific efficacy of pembrolizumab. Overall, survivals for both were not mature, although neither emerging HR was very encouraging. With crossover allowed for cemiplimab after disease recurrence, an OS disappointment will be ascribed to the cross-over design. The latter will be true if the majority of those randomized to placebo will have indeed crossed over, but it will have converted the study into a comparison of early cemiplimab exposure in the adjuvant setting vs. delayed post-recurrence deployment. Given treatment-emergent adverse events leading to cemiplimab discontinuation in 10% of patients compared with only 1% for placebo, including some long-lasting health challenges, and similarly high rates of toxicity with pembrolizumab, including unexplained deaths, a blanket approval for adjuvant use would be inappropriate. But with such an approval likely to be granted by some regulatory agencies, the adverse events and the associated financial toxicities will require individual physicians to decide the best path forward for their patients.

## CAR-T in solid tumors

- There were multiple presentations highlighting the use of chimeric antigen receptor (CAR)-T in solid tumors, all again finding solid tumors far more challenging than their liquid counterparts. In gastric/gastro-esophageal junction (GEJ) adenocarcinoma, Claudin 18.2 has emerged as a target of interest with, zolbetuximab-clzb, a claudin 18.2 (CLDN18.2)-directed cytolytic antibody having received first-line approval in locally advanced unresectable and metastatic gastric/GEJ adenocarcinoma in combination with a fluoropyrimidine and oxaliplatin for patients with HER2-negative tumors and ≥75% of cells expressing moderate-to-strong Claudin 18.2 staining.^32^^,^^33^  **Abstract 4003,** presented by Changsong Qi of Peking University Cancer Hospital in Beijing, China randomized 156 patients (2:1) with advanced gastric/GEJ cancer who had received more than two prior lines of therapy with 250 × 10^6^ cells infused up to 3 times for the 104 randomized to CAR-T therapy targeting Claudin 18.2 (Satri-cel) and either apatinib, paclitaxel, docetaxel, irinotecan or nivolumab given per physician’s choice to the 52 in the TPC arm.^34^^,^^35^ Patients were eligible if they were HER2-negative and had IHC 2+/3+ for Claudin 18.2 in ≥40% of cells, a cutoff that expanded the patient pool compared to prior studies. In the 156 patients comprising the ITT population, the median PFS was 3.25 months with Satri-cel compared to 1.77 months with physician’s choice (HR: 0.366, 95% CI: 0.241-0.557) with median OS of 7.92 vs. 5.49 months, respectively (HR: 0.693, 95% CI: 0.457-1.051). Of the 88 patients treated with Satri-cel, there was 1 treatment-related death and 4 patients with Grade 3 immune effector cell-associated neurotoxicity syndrome. The authors noted these findings were the first randomized controlled trial (RCT) in which CAR-T demonstrated superiority compared with SOC in patients with advanced gastric/GEJ adenocarcinoma expressing Claudin 18.2.^35^ However, the fact that only 88/104 patients received Satri-cel, serious drug-related adverse events occurred in 35% of patients with cytokine release syndrome in 95.5% of patients, albeit Grade 1/2 in 90.9%, and there was a PFS benefit of only 1.5 months without an improvement in OS make this an unpromising option in advanced disease that will require that we wait to see if future studies in the first line randomizing fluoropyrimidine- and platinum-containing chemotherapy against zolbetuximab-clzb, can prove the latter to be truly better.
- **Abstract 102,** presented by Stephen Bagley of the University of Pennsylvania, was a phase 1 study investigating a bivalent CAR-T product against EGFR and IL13Rα2 administered intracerebroventricularly in patients with recurrent glioblastoma multiforme (rGBM).^36^ CAR-T cells were given to 18 patients through an Ommaya reservoir, of which 13 patients had measurable disease prior to treatment initiation. Of these 13 patients, 1 (8%) had a partial response and 8 (61%) demonstrated stable disease, a largely meaningless percentage given a median PFS of 1.9 months. Although median OS was not reached, the median follow-up was only 8.1 months, a short duration even for rGBM. Importantly 100% of patients experienced Grade 1-3 immune-effector cell associated neurotoxicity syndrome (ICANS) but also importantly, there were no grade 4-5 events. Although larger head-to-head studies would be needed to confirm clinical benefit, there are limited other therapeutic options in the rGBM setting. The very limited activity is not surprising in a difficult to treat disease like rGBM. And while the authors argued more work is needed to optimize this treatment strategy, the study’s contribution may eventually lie in having demonstrated the safety of intracerebroventricular administration of bivalent CAR-T cells, a strategy that may attain better outcomes with diseases that are more treatment-responsive.^36^^,^^37^

## Malignant hematology—multiple myeloma

- There were many great presentations from the field of multiple myeloma at ASCO 2025, including long-term follow-up data from the CARTITUDE-1 trial and a phase 1 study of a novel tri-specific antibody in relapsed/refractory multiple myeloma (rrMM). Peter Voorhees of Atrium Health/Levine Cancer Institute presented **Abstract 7507** which updates OS data with 60.4 month median follow-up from the Phase 1b/2 CARTITUDE-1 trial that evaluated cilta-cel, a CAR T-cell therapy engineered to target B-cell maturation antigen (BCMA), expressed at high levels on multiple myeloma cancer cells.^38^^,^^39^ In total, 97 patients received cilta-cel; the median OS was 60.6 months, and 32 patients (33%) are alive at 5 years without disease progression. While 7 of the 32 patients had high-risk cytogenetics and all were described as “heavily pre-treated” with a median of 6.5 prior lines of therapy, one should acknowledge that heavily pre-treated can often be a surrogate for indolent disease that allowed many prior treatments to be administered and could explain the sustained responses with the use of cilta-cel in patients exposed to multiple lines of therapy. It will be intriguing to see if similar prolonged durations of response are observed in follow-up data for the CARTITUDE-4 trial, as cilta-cel moves up in lines of treatment for rrMM.^40^
- Many advances have been made in rrMM as a result of bispecific antibodies targeting BCMA (elranatamab and teclistamab) or GPRC5D (talquetamab) on the surface of plasma cells and CD3 on T-cells. **Abstract 7505,** presented by Niels WCJ van de Donk of Amsterdam University Medical Center in the Netherlands, reports on the phase 1 results of an exciting tri-specific antibody referred to as JNJ-5322, targeting GPRC5D and BCMA alongside CD3, in triple-exposed patients (proteasome inhibitor, Anti-CD38, and Immunomodulatory drugs [IMiDs]).^41^ At the recommended phase 2 dose (RP2D) of 100 mg every 4 weeks, 100% of patients (*n* = 27) who had not received a BCMA or GPRC5D-directed therapy had a response to JNJ-5322, of which 70.4% achieved a CR or stringent CR, and the 12-month PFS was 95%. Of the 36 total patients treated at the RP2D, 33% had grade 3 or 4 infections, and no patients developed ICANS. Of the 147 patients across all dose levels, there were 5 deaths, of which 1 occurred at the RP2D due to treatment-emergent pneumonia. Although larger prospective studies are needed to confirm the efficacy, durability of responses, and safety of JNJ-5322, these results highlight a promising new therapy in rrMM.

## Malignant hematology—myeloproliferative neoplasms

- **Abstract LBA3,** presented by Andrew T. Kuykendall of Moffitt Cancer Center reported the results of the phase 3, double blind, placebo controlled VERIFY trial that studied the addition of rusfertide to SOC phlebotomy ± cytoreductive therapy in polycythemia vera (PV).^42^ Rusfertide is a first-in-class, self-injected, hepcidin mimetic that, when added to SOC, led to more patients not requiring phlebotomy (76.9% vs. 32.9%, *P* < 0.0001), fewer mean number of phlebotomies over 32 weeks from randomization (0.5 vs. 1.8, *P* < 0.0001), and a higher proportion of patients maintaining a hematocrit <45% (62.6% vs. 14.4%, *P* < 0.0001) compared with SOC alone. Rusfertide also led to improvement in symptoms as reported by reductions in PROMIS Fatigue SF-8a and MFSAF TSS7 scores at 32 weeks. The morbidity and mortality associated with PV is typically the result of cardiovascular and thrombotic events due to elevated hematocrits, and with disease transformation, all of which will be monitored for up to 3 years in Parts 1b and 2 of the study.

Taken together, the ASCO 2025 highlights show important advances in therapy for cancer, even as it demonstrated that progress generally continues to be incremental. An often-repeated statement on change from businessman Satya Nani, “A little progress each day adds up to big results” may be apt for oncology therapeutics in 2025.
